# Utterances as Signals for Sharing Tacit Images in Collective Interaction

**DOI:** 10.3389/fspor.2022.851568

**Published:** 2022-06-20

**Authors:** Naoto Shoji, Yasuyuki Hochi, Takuya Ohshiro, Yoshihisa Ono, Motoki Inoue, Motoki Mizuno

**Affiliations:** ^1^Department of Sport and Health Sciences, School of Health Sciences, Asahi University, Mizuho, Japan; ^2^Department of Sports and Health Sciences, Faculty of Physical Education, Japan Women's College of Physical Education, Tokyo, Japan; ^3^Department of Social Work, Faculty of Health and Welfare Human Services, St. Catherine University, Matsuyama, Japan; ^4^Faculty of Health and Sports Science, Juntendo University, Inzai, Japan; ^5^School of Health and Sport Sciences, Chukyo University, Toyota, Japan; ^6^Graduate School of Health and Sports Science, Juntendo University, Inzai, Japan

**Keywords:** social signal, sharing images, knowledge, coaching, team building, leadership

## Abstract

In ball games, individuals collaborate to enhance their team's performance by sharing images and ideas that have not been verbalized. One of a coach's roles is to ascertain whether players share a common understanding of their team's images so as to devise tactics. Accordingly, this study aimed to verify the hypothesis that sharing images such as tacit knowledge that has not been verbalized occurs in collective interaction when utterances increase substantially during problem-solving. The participants were 13 male university handball players whose teams were championship contenders in Japan. A mixed methods research design was employed. Scenes in which two groups engaged in problem-solving were recorded and data of each participant's utterances were obtained. The utterances were analyzed quantitatively by employing Smirnoff-Grubbs and the time periods including those with a substantial number of utterances were identified. What happened during the identified time periods verified as outliers including the high frequency utterances were analyzed qualitatively by employing consensual qualitative analysis. Finally, the results of the consensual qualitative analysis were used to examine statistically to determine whether specific events occurred during times of extreme high frequency utterances. The exact binomial test was used to determine the 95% confidence interval of the population ratio and the effect size (g) of the mother ratio (0.05) to determine whether non-verbalized images such as tacit knowledge were being shared among members. Of the 26 time periods, 22 were supported the hypothesis. Of the time periods with extremely high utterances, the population ratio of the time periods supporting the hypothesis was 0.846 (CI = 0.681–1.00, *g* = 0.80). The results revealed that tacit image sharing occurred when there were a substantial number of utterances. This study demonstrated the possibility that sharing images that have not been verbalized occurs in collective interaction when there is a *hotspot* of utterances.

## Introduction

In ball games, individuals frequently collaborate with one another to enhance their team's performance. Accordingly, employing the shared mental model, which may be defined as “an organized understanding or mental representation of knowledge that is shared by team members” may be beneficial (Mohammed et al., 2000). Furthermore, it is imperative that players share a game plan. The latter may be elucidated as a basic schema that provides the players with a *big picture* (Gershgoren et al., 2016). Social and cognitive components are crucial to realize a coordinated performance in the pursuit of superior collective performances (Eccles and Tenenbaum, 2004). However, the training to share a playing image, creating a shared mental model, or using schema does not occur with explicit communication because such images involve more than verbalized thoughts. The creation of organizational knowledge is one theory that may be employed to explain the process of sharing images between players and coaches. Nonaka (1994) stated, “The process organizationally amplifies the knowledge created by individuals and crystallizes it as part of the knowledge system of an organization.” Furthermore, the process is a continual spiral of tacit and explicit knowledge through the conversion of four modes of knowledge, namely, socialization (tacit to tacit), externalization (tacit to explicit), a combination (explicit to explicit), and internalization (explicit to tacit) (Nonaka, 1994; Nonaka and Toyama, 2003). In ball games, if players have good plans and/or tactics, they try to share their internal resources such as an image and idea with their teammates. In knowledge conversion, while socialization occurs when two or three players endeavor to share tacit knowledge, externalization happens when the players cannot convert their tacit verbalized images and/or ideas to verbalized images and ideas, that is, explicit knowledge. Regarding knowledge sharing, the study showed that there are four channels of knowledge sharing in soccer teams: (1) observing/imitating, (2) peer exchange/peer communication, (3) labor mobility, and (4) knowledge brokers. The study emphasized the positive impact of knowledge sharing in teams on elite player development and performance and the need for future knowledge management tactics to capitalize on the untapped potential of knowledge sharing (Werner, 2018). It has also been shown that coach humanities are associated with player creativity, with knowledge sharing as a central outcome (Tuan, 2020), and it is becoming shared that knowledge sharing in sports teams is a key issue in elite sports. Then, it was suggested that the congruence of the mental models of leaders and followers may improve the creative performance of followers, regarding the type of experiences, nature of outcomes, and emotions used in the sharing of visions and messages. Mental model congruence has been shown to be potentially important in team sports as well (Griffith et al., 2018). One of coaches' roles encompasses ascertaining which mode of knowledge the players are utilizing. However, this may be difficult because it is dependent on their personal skills, for example, tacit knowledge, sense, and instinct so as to understand the context of the situation. It is imperative that coaches determine whether players share a common understanding of their teams' images for devising tactics in a game and a training.

A multitude of concepts may be needed for understanding coaches' roles, including knowledge creation, social signals, and honest signals (Nonaka, 1994; Pentland, 2008). Weiss (2021), for example, in the context of communication in sport, also focus on words as symbols or signs, as well as attitudes and words as responses to unconscious meanings. We predicted unconscious behavior was a further key concept because players and coaches do not communicate verbally to convey that they understand others' images and ideas. Another important factor to consider is the time horizon. In other words, when considering the sharing of images and ideas within a team, conventional research on knowledge creation for sports teams has assumed knowledge creation and knowledge management over a relatively long span of time related to business administration. However, especially in elite on-the-field sports, dynamic and improvisational knowledge creation may be required in a very short period of time depending on the situation. In the execution of such improvisational knowledge creation, it is imperative for coaches to be able to ascertain whether players really understand and are able to share their team's tacit images and ideas with their teammates.

Accordingly, we quantified the number and length of utterances in small group activities as a part of the team building training. Subsequently, we found the time period of utterances increased substantially and many individuals repeated short utterances frequently. These utterances lasted <1 s. During this particular time period, there were many more utterances in comparison to other time periods. Thus, we hypothesized that increasing the number of utterances substantially is a social signal that conveys tacit image sharing. Therefore, this study aimed to examine whether sharing tacit images and ideas that have not been verbalized occurs when utterances increase substantially in collective interaction.

## Methods

### Research Design

**Figure 1:**
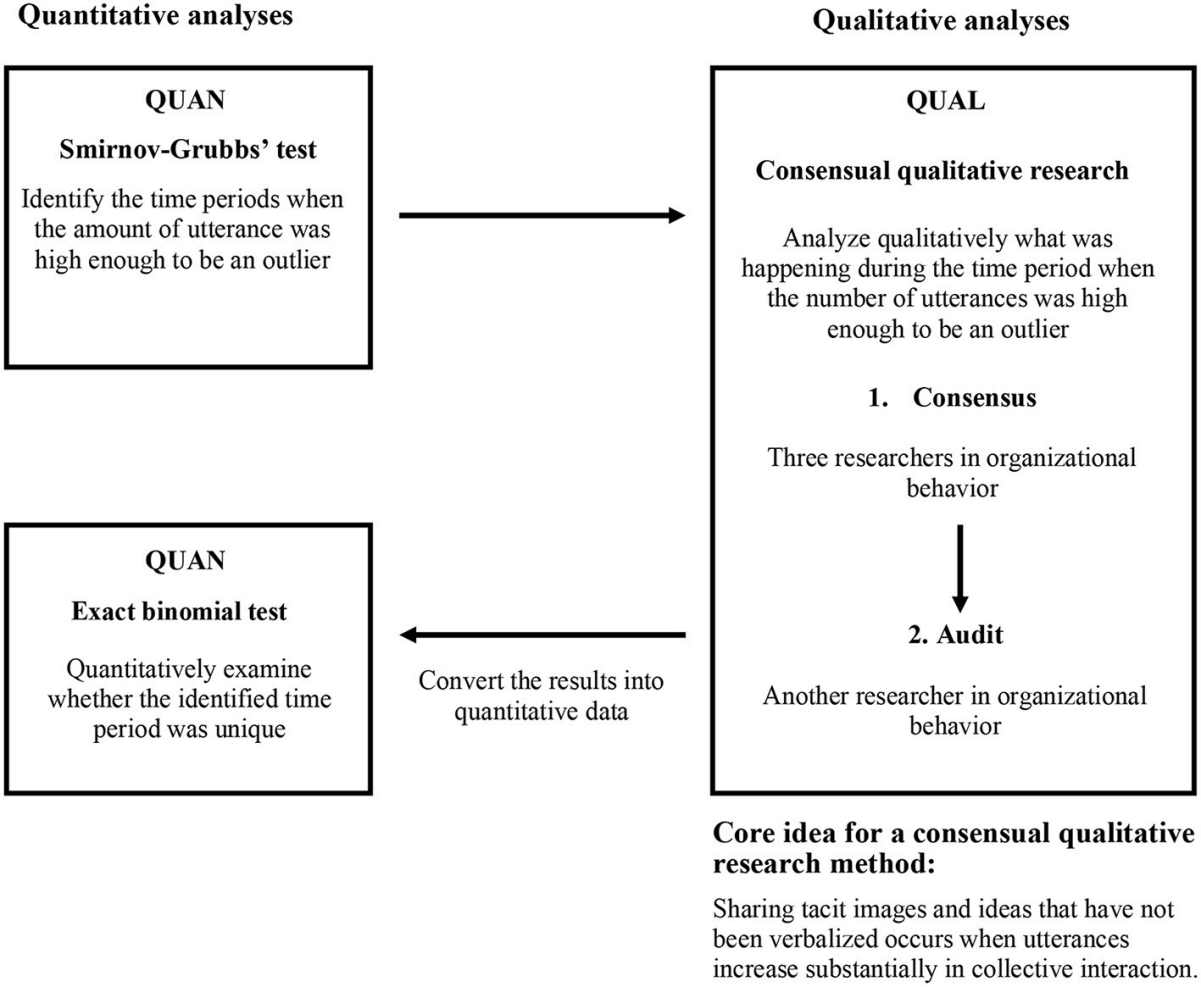
Flow of the mixed methods research, QUAN + QUAL + QUAN design in the Convergent model.

A mixed methods research design was used. As noted previously, the purpose of the study was to examine whether a substantial increase in the number of utterances was a social signal to convey the sharing of tacit images in collective interaction. For achieving the above purpose, while quantitative methods, specifically, Smirnoff-Grubbs and the exact binomial test were employed to determine the number of utterances, a qualitative method, namely, the consensual qualitative research method was used to analyze what occurred in the situations (Figure 1).

The design choice was QUAN+QUAL+QUAN (Three-stage equal-status concurrent design) in a triangulation design Convergent model and illustrated in Figure 1 (Schoonenboom and Johnson, 2017). In the Convergent model, quantitative and qualitative data are given equal importance (Creswell and Clark, 2017; Levitt et al., 2018). Data of the present study are acquired simultaneously and the research purpose is only achieved when the results of all three analyses are combined. The reasons for adopting Mixed-Methods in this study and the basic analytical strategy were as follows. In order to verify from a qualitative analysis perspective whether the sharing of implicit images and ideas is enhanced when there is a statistically extreme increase in the number of utterances in group interactions, this study first rigorously identified the time periods during which extremely high utterances were detected that could be described as quantitative outliers or anomalies by combining multiple statistical (the Smirnoff-Grubbs analysis, 95% CI calculation of the average number of utterances and 95th percentile calculation of the number of utterances were employed). The time periods identified as extremely high in speech were then qualitatively examined to determine what was happening there (the Consensual qualitative research was employed). Finally, the results derived from the consensus qualitative research method were converted into quantitative data to test quantitatively whether the sharing of implicit images and ideas was more advanced when extreme utterances were identified (employed the exact binomial test). This allowed us to integrate the scientific basis for (1) the fact that the target of this study was a time period when the number of utterances was high enough to be an outlier (QUAN), (2) what events were occurring during that time period (QUAL) and (3) the fact that certain events were observed during the target time period (QUAN). The above flow allowed the statistical analysis of the quantitative data and the analysis of the qualitative data to converge their respective results into a single result, which revealed what was happening at the singular point of extreme speech frequency and provided insight into the process of the sharing of images and ideas in a sports team.

### Participants

The participants were 13 male university handball players who were selected randomly from 34 players in two teams that were championship contenders in Japan (Table 1). Their mean age was 18.77 years (SD = 0.93, Range = 18–21). Two locations were set up in advance and the players were given the freedom to sit where they wanted to in the group. The group that sat in pre-selected seats was selected as participants.

**Table 1:** Participants of this study.

	**Participants of group A**							**Participants of group B**					
	**A**	**B**	**C**	**D**	**E**	**F**	**G**	**H**	**I**	**J**	**K**	**L**	**M**
1st trial	✓	✓	✓	✓	✓	✓		✓	✓	✓	✓	✓	
2nd trial	✓		✓	✓	✓	✓	✓	✓	✓	✓	✓	✓	
3rd trial	✓	✓	✓	✓	✓	✓		✓	✓	✓	✓	✓	✓

### Data Collection

**Figure 2:**
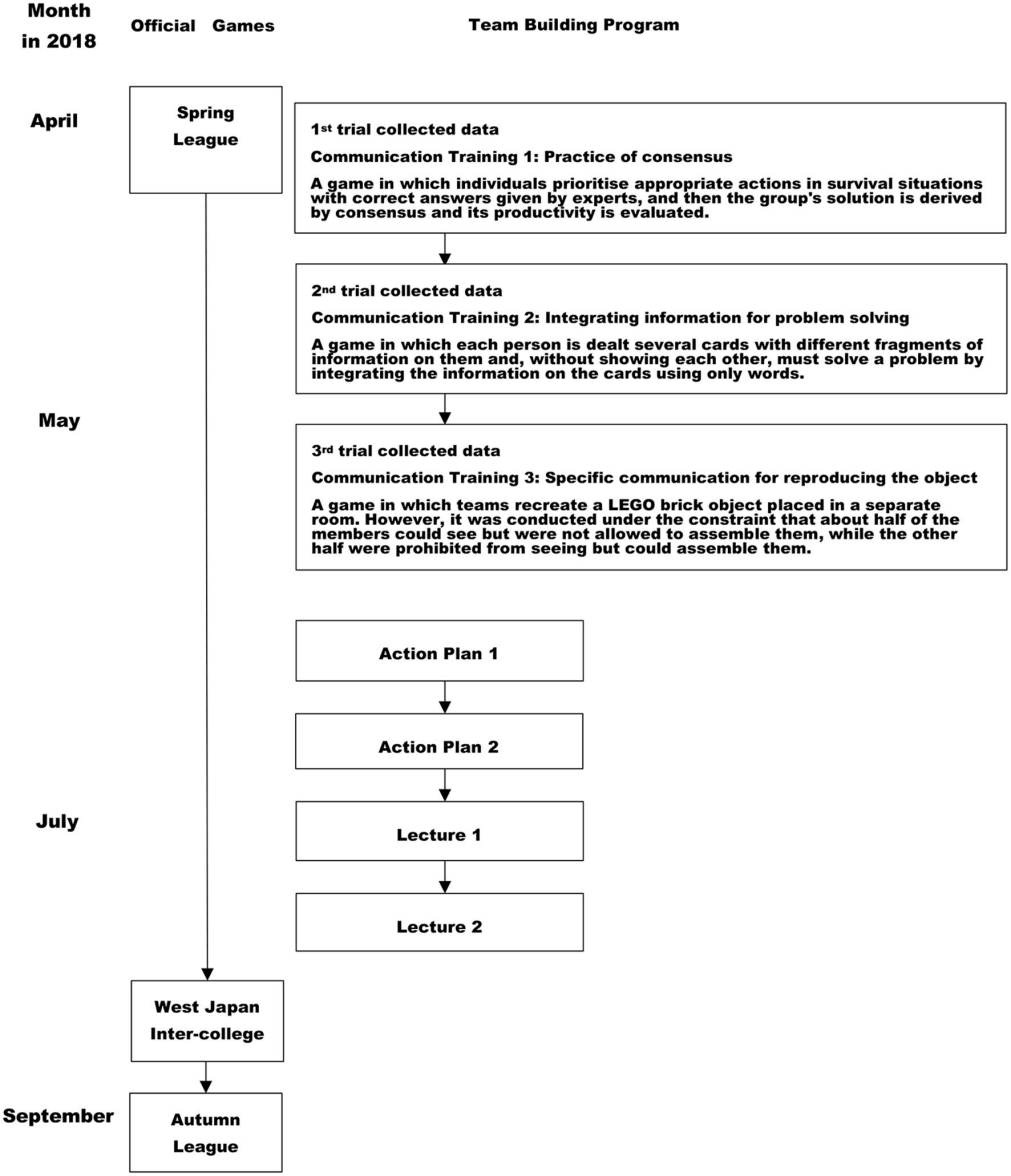
Trials in the team building training.

The data were collected during team building training from April to May, 2018 (Figure 2). Small group activities during the team building training were employed to collect utterances data and video cameras were used to record problem-solving scenes. Both groups' problem-solving situations were each recorded three times, thus obtaining a total of six pieces of video data. Each problem-solving scene lasted between ~24 and 36 min. Three trials of team building training program were targeted for this study (Figure 2). An overview of the issues that the subjects addressed in each trial of the data collection is presented in Figure 2. The themes of each trial are as follows. The 1st trial aimed to practice of consensus for problem-solving which required correct consensus, and each group were assessed group performance effectiveness. The assessment aimed feedback for participants' learning, it did not aim to obtain data for this study. The 2nd trial aimed to practice of integrating information for a problem-solving game which participants were required to integrate fragmentary information each participant held for solving tasks. The 3rd trial aimed to practice to specify for problem-solving which required specific communication for reproduce the object with “LEGO”. A common feature of the tools used in each trial was to be required to exhibit participants' creativity and interaction skills. Facilitator was first author who had extensive experience in facilitating team building and other forms of collaboration. Two analysts watched the video data and recorded each participant's utterances in one second increments in a matrix. Rigor was ensured by double-checking the data of the utterances, with two people watching the same video. Time was depicted on the vertical axis and the subject code on the horizontal axis. The number of utterances as well as the duration of each participant's utterances was quantified.

### Data Analysis

The data of five of the six videos were analyzed. In the video that was excluded, the participants were moving while trying to solve a problem and two participants overlapped in a video. It was deemed that the utterances data may have included inaccurate utterances and thus, was excluded. That was a technical limitation.

Data analyses were conducted by Mixed Methods, which combines quantitative observational studies with qualitative data analysis. All data were collected and analyzed cross-sectionally. In addition to quantifying the perceivable information of the number of utterances (QUAN), deep insight using qualitative data is necessary to clarify what was happening at singularities with extremely high utterances (QUAL). At the same time, quantitative analysis was added to the analysis protocol to clarify the extent to which specific phenomena occur at times of extremely high utterances obtained from qualitative data (QUAN).

First, the small group problem-solving sessions were divided into 15-, 30-, and 60-s segments based on the matrix in which each participant's utterance data were recorded in 1 second increments. Thereafter, descriptive statistics for each segmented time period were calculated. Specifically, the total number of utterances, mean number of utterances (95% CI), range of utterances, and 95th percentile of utterances for each session were calculated.

Second, periods when there were extremely high speech rates for 15, 30, and 60 s were identified. Smirnoff-Grubb's analysis was employed to determine the number of utterances that statistically were outliers. In this study, outliers were defined as the number of utterances that exceeded the 95th percentile for each session and were found to be significantly different.

Third, consensual qualitative analysis (Hill et al., 2005; Hill, 2021) was employed to analyze what was happening in the small groups during the outlier periods from the perspective of collective interaction. CQR is ideal because it involves a rigorous method that allows several researchers to examine data and come to consensus about their meaning, thus reducing the biases inherent with just one person analyzing the data (Hill et al., 2005). Consensual qualitative research was considered the most suitable method for this study because the present study challenge was to eliminate researcher bias as much as possible in order to test and present hypotheses. Four researchers specializing in organizational behavior and organizational psychology were engaged in the qualitative analysis. One of them was involved in the analysis process as an auditor; the age range of the four researchers was diverse, ranging from their 30 to 50 s; one of the three analysts had national championship-level experience as an athletic coach, and his perspective from the athletic field was included; and all four were familiar with the theoretical framework of this study, “knowledge creation,” which ensured sufficient robustness. In addition, the four analysts were well trained in qualitative data analysis and had extensive experience analyzing qualitative data. The basic protocol of the analysis was based on the work of Hill et al. and consisted of presenting the core idea, followed by a multi-person collegial discussion, and then Auditing by the Auditors (Hill et al., 2005; Hill, 2021). As part of the analysis protocol for the congruent qualitative research method, the three analysts first watched the video to be analyzed repeatedly in their respective offices individually to avoid peer pressure, and wrote on a spreadsheet what was happening during the time period covered (the time period indicated on the joint display). The analysts were also free to check the context before and after the video. The three analysts then met in the same space on a different day, watched the videos together, discussed what was happening during that time period, and agreed whether or not what was happening during each time period constituted the sharing of the SECI model, as hypothesized. The results of the discussion were then brought to the auditor, who himself evaluated what was happening during that time period by looking at the target video, and assessed whether the hypotheses were consistent with what was happening on the video. Although the responsible author was present at that time, he was committed to answering the auditor's questions regarding the confirmation items and did not participate in the analysis. In addition, we examined the appropriateness of the wording of the qualitative outputs included in the results of the discussions and made the necessary modifications to make the descriptions more accurate.

Finally, the results of the consensual qualitative analysis were used to examine statistically whether specific events could have occurred when the number of utterances was substantially high. Specifically, the exact binomial test was used to determine the 95% confidence interval of the ratio and the effect size (g) of the mother ratio (0.05) to determine whether non-verbalized images such as tacit knowledge were being shared among the members when the number of utterances was substantially high.

### Ethical Considerations

This study conducted in accordance with the World Medical Association's Declaration of Helsinki. And, the study was approved by the research ethics committee of the department to which the first author belonged (approval number 2017017).

## Results

First, the data on the utterances were analyzed quantitatively and the time periods when the number of utterances was high enough to be an outlier were identified. Subsequently, a qualitative analysis was conducted to determine what was happening during periods of substantially high utterances, which were derived from the quantitative results. The main results of the mixed analysis were presented as a joint display.

The total number of utterances in each session, average number of 15-, 30-, and 60-s utterances (95% CI), range of utterances, and 95th percentile value of utterances were calculated (Table 2). The 95% confidence intervals for the mean number of utterances were narrow for all sessions and all time slices, thus indicating that there were no large fluctuations in the number of utterances during the entire session. Thereafter, Smirnoff-Grubbs analysis was conducted to identify 32 time periods in which the number of utterances exceeded the 95th percentile, which were statistically outliers and considered to have a substantial high number of utterances (Table 3). Three researchers conducted a qualitative assessment of the 32 time periods and decided that it would be appropriate to exclude six of these time periods from the analysis because the data were collected before the group problem-solving session started. A total of four researchers (three researchers and one auditor) conducted a qualitative analysis of what happened in the 26 time periods. The results revealed that the study hypothesis was supported in 22 time periods (84.6%). In relation to the other four time periods, it is possible that the characteristics of the task on which the small group was working (time limit, impending end time) increased the number of utterances and the hypothesis could not be supported (Table 3). In addition, the researcher who conducted the audit proposed a new hypothesis: number of utterances increase when conflicts or conflicting alternatives are evident. The hypothesis was proposed because images of a concrete space were shared in the four time periods even though they appeared to be mere information exchanges.

**Table 2:** Descriptive statistics of utterances.

**Group**	**Trial**	**Time period (s)**	**Number of time periods**	Number of utterances^*^	**Average number**	**SD**	**95% CI**	**Range**	**95th percentile value**
Group A	1st trial	15	111	351	3.16	1.51	2.88–3.45	1–8	6.00
		30	56	310	5.54	2.3	4.92–6.15	1–12	10.15
		60	28	292	10.43	2.7	9.38–11.48	4–17	15.65
	2nd trial	15	136	450	3.31	1.48	3.06–3.56	0–7	6.00
		30	68	420	6.18	2.21	5.64–6.71	2–13	10.00
		60	34	401	11.79	2.95	10.76–12.82	5–20	17.00
	3rd trial	15	112	361	3.22	1.7	2.91–3.54	0–9	6.35
		30	56	329	5.88	2.74	5.14–6.61	0–13	12.15
		60	28	316	11.29	4.34	9.60–12.97	3–20	19.55
Group B	2nd trial	15	143	623	4.36	2.54	3.94–4.78	0–11	8.80
		30	71	584	8.23	4.26	7.22–9.23	0–17	16.00
		60	35	569	16.26	7.62	13.64–18.87	0–31	27.80
	3rd trial	15	96	415	4.32	2.31	3.86–4.79	0–12	8.00
		30	48	390	8.13	3.71	7.05–9.20	0–17	14.55
		60	24	376	15.67	5.64	13.29–18.05	6–26	25.40

^*^Total numbers of utterances decreased as time increased to 15, 30, and 60 s. Several utterances crossed a border of multi time periods, in the case, the utterances counted as both time periods, hence, double counted utterances decrease as time periods became longer.

**Table 3:** Joint display showing that a substantial number of utterances signaled the sharing of an image.

Outlier analysis*of time periods, including number of utterances exceeding 95th percentile										**Qualitative assessment of what happened in the hotspot of utterances**						**Exact binomial test**
**Group**	**Trial**	**Time period**	**Number of observed utterances**	**Mean**	**Variance**	**T**	**d**	***p***	**Deviation**	**Case**	**Time period: min:sec**		**Results of qualitative assessment/What was happening**	**Proved hypothesis**		
											**Start**	**End**		**Researchers**	**Auditor**	
Group A	1st trial	15	8 (2)	3.16	2.28	3.20	109	0.001	82.05	1	4:30	4:45	Members tried to hear their opinions after sorting out information. They gave one another alternatives.			
										2	31:45	32:00	Members experienced time constraints and stimulated discussions to reach a consensus quickly. They reached a final answer.	✓		
		30	12 (1)	5.54	5.27	2.82	54	0.003	78.09	3	4:30	5:00	Members tried to hear their opinions after sorting out information. They gave one another alternatives. Conflicts emerged.			
			11 (1)	5.42	4.58	2.61	53	0.006	74.26	4	31:30	32:00	Members experienced time constraints and stimulated discussions to reach a consensus quickly. They reached a final answer.	✓		
	2nd trial	15	7 (2)	3.31	2.20	2.49	134	0.007	74.93	6	11:45	12:00	Members shared a basic framework (schema) for problem-solving. They experienced hope and felt positive. They created and shared new knowledge. They discussed where they would obtain answers from in situations in which they did not know anything. During this time, sharing images progressed and they arrived at a consensus.	✓	✓	Success 22 Non Success 4 Population ratio 0.846 95%CI = 0.681–1.00 ES(g) = 0.80 The number of outlier utterances all showed high deviation values (68.32–84.00), the fact was the evidence indicating a time period with an extremely high number of utterances. It can be said that image sharing was going on in most of these time periods. It can be said that an extremely high number of utterances was a signal that image sharing was in progress.
										7	12:30	12:45	Members integrated information, and they obtained the final answer. They modified some alternatives, which emerged in an answer.	✓	✓	
		30	13 (1)	6.18	4.89	3.08	66	0.001	80.86	8	11:30	12:00	Members integrated the information they held, and they created shared common images needed for problem-solving.	✓	✓	
			11 (1)	6.07	4.25	2.39	65	0.010	72.31	9	14:30	15:00	One member provided an important clue to the solution, and members shared a clearer image of the path to the solution. That led to the solution, which elated the “Ba” very much.	✓	✓	
		60	20 (1)	11.79	8.71	2.78	32	0.005	77.83	10	11:00	12:00	Members were progressing to organize and integrate the fragmented information held by them. They created shared images needed for problem-solving.	✓	✓	
	3rd trial	15	9 (1)	3.22	2.88	3.41	110	0.000	84.00	Excluded	1:00	1:15	These were excluded from the analysis because it occurred before members tackled the problem-solving. However, an analyst noted they were trying to share difficult “Japanese kanji” orally and that this also constituted image sharing.	–	–	
			8 (1)	3.17	2.60	3.00	109	0.002	78.41	Excluded	0:30	0:45		–	–	
			7(3)	3.13	2.41	2.50	108	0.007	72.76	Excluded	0:45	1:00		–	–	
										11	12:30	12:45	Members created visual information,which only one member verbalized and they progressed to create the shared image.	✓	✓	
										12	13:30	13:45	A few members verbalized visual information verbalize. The remaining members visualized the verbalized information and externalized explicit words. Members converted visual information into explicit information and started sharing one image.	✓	✓	
		30	13 (2)	5.88	7.49	2.60	54	0.006	75.99	Excluded	0:30	1:00	These were excluded from the analysis because it occurred before members tackled the problem-solving. However, an analyst noted they were trying to share difficult kanji orally and that this also constituted image sharing.	–	–	
										Excluded	1:00	1:30		–	–	
		60	20 (1)	11.29	18.80	2.01	26	0.027	70.07	Excluded	0:00	1:00		–	–	
Group B	2nd trial	15	11 (1)	4.36	6.47	2.61	142	0.005	76.14	13	24:45	25:00	Members progressed with the externalization of already shared image.	✓	✓	
			10 (2)	4.31	6.20	2.28	141	0.012	72.40	14	7:00	7:15	Members confirmed a shared image, suggested an unclear point, and created a shared image based on information each member had.	✓	✓	
										15	16:30	16:45	Members were confirming and sorting out information, which each member recognized. They created shared image between them.	✓	✓	
			9 (4)	4.23	5.82	1.98	139	0.025	68.78	16	3:15	3:30	A member who possessed many words shared an image.	✓	✓	
										17	5:45	6:00	Members progressed to create a shared image based on information each member had.	✓	✓	
										18	15:15	15:30	Members progressed to create a shared image by integrating information they had.	✓	✓	
										19	15:45	16:00	Members were sorting and integrating multi information, and an answer (shared image) become clear.	✓	✓	
		30	17 (1)	8.23	18.18	2.06	69	0.022	70.59	20	16:30	17:00	Members were confirming and sorting information, which each member recognized. They created a shared image between them.	✓	✓	
		60	31 (1)	16.26	58.02	1.94	33	0.031	69.34	21	15:00	16:00	Members progressed to create a shared image by integrating fragmented information they had. They were sorting and integrating multi information, and an answer (shared image) become clear.	✓	✓	
	3rd trial	15	12 (1)	4.32	5.34	3.32	94	0.001	83.25	22	5:15	5:30	Members progressed to share images of arrangements and objects for problem-solving with language and gestures.	✓	✓	
		30	17 (1)	8.13	13.73	2.40	46	0.010	73.91	23	5:00	5:30	A member gave visual arrangements information obtained to other members. Members progressed to share an image of arrangements and objects for problem-solving with the visual arrangements information.	✓	✓	
			15 (1)	7.94	12.28	2.02	45	0.025	69.03	24	7:30	8:00	Members felt anxiety to achieve because they each had a limited role. However, after members shared confirmed role authority and methodology, they went to problem-solving.	✓	✓	
		60	26 (2)	15.67	31.80	1.83	22	0.040	68.32	25	5:00	6:00	A member gave visual arrangements information obtained to other members. Members progressed to share an image of arrangements and objects for problem-solving with the visual arrangements information.	✓	✓	
										26	7:00	8:00	Members believed they each had a limited role, had differences about the drawn image, and were anxious to achieve. However, once members shared confirmed role authority and methodology, they went to problem-solving.	✓	✓	

*Smirnov-Grubbs test (One-side test).

In order to verify whether the hypothesis was supported quantitatively in 22 out of the 26 time periods analyzed, the exact binomial test was employed to test the population ratio, which was found to be significant (*p* < 0.000). The results showed robust evidence in support of the hypothesis that image sharing occurs when there are a substantial number of utterances [0.846, 95%CI = 0.682–1.00, ES(g) = 0.80 Table 3].

## Discussion

Analyses of the study revealed that the study hypothesis was supported. Thus, a substantial number of frequent utterances may indicate that a number of participants had the same image in mind in the scenes. In sports training, it is most likely that a shared image has been formed among players when there is a substantial increase in utterances and on the contrary, more dialogs may be needed if there is no increase in the number of utterances. It would be most beneficial for coaches to be able to ascertain this objectively. This is an important because it is difficult to do things alone and collaboration is required in many situations in sports.

The results of this study can help coaches to determine whether images and ideas that are not verbalized were shared among the players. Coaches may conclude that increased utterances indicate that images have been shared among players. If utterances do not increase, coaches should explain the notion of images and ideas about tactics, strategies, feelings, senses, and tacit knowledge to players. In such situations, the team may not have basic schema, shared mental model, or shared cognition related to a game plan (Mathieu et al., 2000; Zhou and Wang, 2010). Pentland (2010) asserted that human behaviors can be predicted easily if honest signals are used. This study presented, if coaches observe a substantial increase in utterances as a signal, this will enable them to understand the team's status in relation to understanding and sharing a game plan that does not include verbalized ideas (Pentland, 2010).

The results of this study indicate that a substantial number of utterances in collective interaction may be a social signal that tacit images may be about to be converted into formal knowledge. This may include the conversion of tacit images into explicit knowledge, namely, the externalization stage of the SECI model in the knowledge creation theory (Nonaka, 2019). In other words, we thought substantially increasing of utterances occurred in the step of “externalization” of the concept of knowledge conversion. Because, participants progressed to create shared image based on someone's dim image with verbalizing, when substantial increasing of utterances occurred in participants' interaction. Externalization is the process of articulating tacit knowledge into explicit knowledge. When tacit knowledge is made explicit, knowledge is crystallized, thus allowing it to be shared by others. It then becomes the basis of new knowledge (Nonaka, 2019, 2021). Concept creation in new product development is an example of this conversion process. A quality control circle, which allows employees to make improvements to the manufacturing process by articulating the tacit knowledge accumulated on the shop floor after years of doing the job, is another example. The successful conversion of tacit knowledge into explicit knowledge depends on the sequential use of metaphor, analogy, and model (Nonaka et al., 2000). The latter is very similar to the process of creating new ideas during problem-solving in small group activities or creating new playing images and tactics in sports situations. In these situations, metaphorical expressions, analogies, and models are often employed to reconcile images (Nonaka et al., 2000). Nonaka and Konno (1998) argued the place proceeded knowledge conversion is “Ba”, “Organaizing ba is the world where individuals share feelings emotions experiences, and mental models.” That is the same as what coaches and players are required in sports. The concept of knowledge creation is applied to the US. Marine Corps' studies, applying the concept of knowledge creation to sports studies may be valid (Nonaka and Uno, 2020). Nonaka and Konno (1998) asserted that exchanging tacit knowledge is self-transcendental process in which individuals share feelings, emotions, experiences, and mental models. The results of this study revealed that participants shared images, ideas, and feelings, similar to a mental model, in the process of problem-solving. Moreover, an important practice within the SECI model is the translation of specialists' highly personal and/or highly professional knowledge into explicit forms that are easy to understand in the externalization stage of the SECI model (Nonaka and Konno, 1998). This is similar to a coach's role for sharing a game plan. Gershgoren et al. (2016) presented “Game philosophy included the association between shared mental model and (a) tactical under- standing, (b) agreement between the coaches and the players, and (c) agreement among players. On that matter, the coaches postulated that despite them determining the game plan, the players are the ones who eventually implement it. Therefore, the extent of agreement between the players and the coaches, and among the players, is essential for producing coordinated efforts (Gershgoren et al., 2016)”.

In fact, it can be read that conflicts occurred in Cases 3 and 5, among the time periods in which the number of utterances increased extremely (see Table 3). Case 3 was a time period in which conflicts occurred as a result of members sharing their opinions with each other. In Case 5, a conflict had occurred and the members were discussing how to resolve the conflict. However, it can be read from Table 3 that of the 26 cases discussed in this study, these were the only two cases in which conflicts were observed. And of the 26 target time periods derived by the congruent qualitative analysis method, 22 of them showed events that supported the hypothesis of this study, and the effect size (g) was 0.80, an extremely high effect size. Then, in reality, the video data clearly show smiles and joy in many cases. It was inferred that perhaps this expression of emotions such as smiles and joy was due to empathizing with others. It also seems possible that an increase in utterances may occur in order to resolve conflicts, but the fact is that in the data of this study, there were few situations in which the number of utterances increased in order to resolve dissatisfaction or conflicts. This is accepted as a hypothesis and should not be rejected at this point, but should be verified through follow-up studies.

In this study, we think there were special time periods. Those were called the *hotspot* of utterances that were included very high number of utterances. The frequency of the utterances observed during the time period was statistically estimated to be an outlier. The *hotspot* of utterances appeared when individual tacit image was shared between many members in the collective interaction. This resulted in the externalization of the SECI model. In *hotspot* of utterances, tacit images and ideas that were not verbalized were shared. During such moments, players displayed a cheerful, agreeing, and satisfying attitude. The increasing of utterances independent of communication skills, that was thought depend on pleasure of sharing image, and comfortableness of sympathy. In other word, the increasing of utterances was natural unconscious reaction with pleasure and comfortableness. We are of the view that the responses and emotions may include *aha!* experiences that resulted from understanding others' images and ideas that were not verbalized. Thagard and Stewart (2011) revealed that the *aha!* experience is not just a side effect of creative thinking, but rather a central aspect of identifying those ideas that are potentially creative. Therefore, *aha!* experiences in small group activities are a social signal of the demonstration of creativity.

Although many studies have been conducted on group interaction, many studies have excluded very short utterances such as one or two words as they have considered them to be inappropriate for analysis. Moreover, with the exception of non-verbal social interaction most studies have regarded very short utterances as having no meaning (Rimé, 1982). However, in this study, we treated very short words of <1 s such as *yes* and *it* as having a significant mean. In fact, human behavior has been explained simply by unconscious non-linguistic behavior (Buchanan, 2009). The U.S. Marine Corps (2018) noted that implicit communication, that is, communicating through mutual understanding by using a minimum of key, well-understood phrases and/or even anticipating each other's thoughts is a faster and more effective way of communicating than through the use of detailed, explicit instructions.

In this study, participants' reaction and attitude in the *hotspots* concurs with Danek and Wiley (2017) who suggested that the *aha!* experience could be linked to the joy of discovery, confidence in being correct, and a feeling that the solution appeared instantly. We are of the view that participants were connected by sharing their images, which generated happiness, excitement, surprise, and the *aha!* feeling. Sharing images with others may have resulted in feelings of delight and the series of short words may have been induced by happiness, excitement, surprise, and the *aha* feeling. These short expressions may have been an unconscious human response. Emotions expressed in short words should also be measured with a device such as a socio meter, which can measure honest signals from humans (Pentland, 2008; Mizuno et al., 2015). This study's qualitative analysis also revealed that very short words clearly included happiness and excitement. We assumed that participants' happiness and excitement of sharing images triggered many very short utterances, the hotspot occurred as the result.

On the other hand, this study and hypothesis focus only on the time periods when speech is unusually active. While this study shows that implicit images and ideas are shared in the Hotspot of speech, it does not indicate whether implicit images and ideas are being shared at other times of the day. And the possibility that the study focused only on some Hotspots must be considered. First, it is important to focus on what is happening at the singularity. In this study, we focused on singularities (Hotspots) with extremely high utterances. The average number of utterances and their 95% CIs do not show a very wide range, and statistical evidence suggests that the time period we focused on is an outlier or an outlier (see the Table 1). The number of utterances during normal times is not so large. We thought that the *Hotspot* (the moment when an image is shared or immediately after) would appear when the accumulation of these normal times came to fruition. We defined a Hotspot as a short period of time that includes the moment when an image is shared, although it is thought that image sharing occurs over time.

In this study, it is not possible to know whether image sharing is also taking place at other times than the Hotspot. It is possible that image sharing may occur outside of Hotspots, but it is also possible that image sharing is extremely high in Hotspots. However, we confirmed that image sharing was progressing at an extremely high rate at *Hotspot*. This is an important fact that was shown to be statistically significant [*ES(g)* = *0.80*, see the Table 3]. And while it did not appear from the video that image sharing was occurring when the number of utterances was normal, that verification is outside the scope of this study. This study is only to verify what was happening at times when the number of utterances was so extreme that it could be called a singularity. Most importantly, it is significant that even if image sharing did occur at times other than Hotspot, it was clear that a specific phenomenon was occurring: image sharing with an extremely high probability at times of abnormally high utterances.

In recent years, sport performance has come to be viewed as a dynamic system, and attempts have been made to apply the concepts of dynamic systems theory to the study of game structure and the emergence of tactical patterns in team sports (Filho, 2018; Delshab et al., 2020; Lines et al., 2022). Thus, understanding the process of play construction and learning in team sports is an essential study in the pursuit of effective performance optimization. The results of this study suggest that frequent occurrence of short utterances is likely a sign that some image has been shared (the same picture) among the members. Therefore, when multiple members collaborate on a single play in sports, this may provide a clue that images are being shared. If this is the case, it should be a valuable sign for players and coaches. The appearance of an extremely high number of short utterances can be a clue as to whether the work to share the image is sufficient or whether more work needs to be done.

The results help recent research on sensor-based communication. The *hotspot* of utterances was a signal that many people shared the tacit images and ideas. In sensor-based studies, researchers cannot obtain the contents of conversations because of ethical considerations about privacy and personal information (Mizuno et al., 2015). Thus, it was not understood what really happened. However, the results of this study suggest that researchers can understand players in a team share images and ideas without hearing the contents of conversations.

## Conclusion

This study demonstrated the likelihood that images that have not been verbalized are shared in collective interaction when a *hotspot* of utterances occurs.

## Data Availability Statement

The raw data supporting the conclusions of this article will be made available by the authors, without undue reservation.

## Ethics Statement

The studies involving human participants were reviewed and approved by Research Ethics Committee of Department of Health and Sport Sciences, School of Health Sciences, Asahi University. The patients/participants provided their written informed consent to participate in this study.

## Author Contributions

NS planed this study, conducted the analyses, and wrote the manuscript. YH conduct the qualitative analysis as an auditor. He contributed greatly to verbalizing the collective interaction. TO and YO were responsible for the qualitative analysis. MI was responsible for the experimental settings throughout this study. MM played a central role in revising the discussion. All authors contributed to the article and approved the submitted version.

## Funding

This study was supported by 2019 Miyata research grant A of Asahi University (A 19023).

## Conflict of Interest

The authors declare that the research was conducted in the absence of any commercial or financial relationships that could be construed as a potential conflict of interest.

## Publisher's Note

All claims expressed in this article are solely those of the authors and do not necessarily represent those of their affiliated organizations, or those of the publisher, the editors and the reviewers. Any product that may be evaluated in this article, or claim that may be made by its manufacturer, is not guaranteed or endorsed by the publisher.
